# Artificial Room‐Temperature Ferromagnetism of Bulk van der Waals VSe_2_

**DOI:** 10.1002/advs.202504746

**Published:** 2025-05-30

**Authors:** Jinhyoung Lee, Gunhyoung Kim, Hyunho Seok, Hyunbin Choi, Hyeonjeong Lee, Seokchan Lee, Geonwook Kim, Hyunho Kim, Seowoo Son, Sihoon Son, Dongho Lee, Hosin Hwang, Hyelim Shin, Sujeong Han, Geumji Back, Alexina Ollier, Yeon‐Ji Kim, Lei Fang, Gyuho Han, Goo‐Eun Jung, Youngi Lee, Hyeong‐U Kim, Kenji Watanabe, Takashi Taniguchi, Wonjun Shin, Suraj Cheema, Andreas Heinrich, Won‐Jun Jang, Taesung Kim

**Affiliations:** ^1^ School of Mechanical Engineering Sungkyunkwan University (SKKU) Suwon‐si Gyeonggi‐do 16419 South Korea; ^2^ Center for Quantum Nanoscience Institute for Basic Science (IBS) Seoul 03760 South Korea; ^3^ Department of Semiconductor Convergence Engineering Sungkyunkwan University Suwon 16419 South Korea; ^4^ SKKU Advanced Institute of Nanotechnology (SAINT) Sungkyunkwan University Suwon 16419 South Korea; ^5^ Department of Nano Science and Technology Sungkyunkwan University Suwon 16419 South Korea; ^6^ Park Systems Corporation 109, Gwanggyo‐ro, Yeongtong‐gu Suwon‐si Gyeonggi‐do 16229 South Korea; ^7^ Semiconductor Manufacturing Research Center Korea Institute of Machinery and Materials (KIMM) Daejeon 34103 South Korea; ^8^ Nano‐Mechatronics KIMM Campus University of Science & Technology (UST) Daejeon 34113 South Korea; ^9^ National Institute for Materials Science Namiki 1‐1 Tsukuba Ibaraki 305‐0044 Japan; ^10^ Research Laboratory of Electronics Massachusetts Institute of Technology Cambridge MA 02139 USA; ^11^ Department of Electrical Engineering and Computer Science Massachusetts Institute of Technology Cambridge MA 02139 USA; ^12^ Department of Materials Science and Engineering Massachusetts Institute of Technology Cambridge MA 02139 USA; ^13^ Department of Physics Ewha Womans University Seoul 03760 South Korea; ^14^ Department of Nano Engineering Sungkyunkwan University Suwon 16419 South Korea

**Keywords:** ferromagnetic, magnetic force microscopy, nano‐crystallization, van der Waals materials, vanadium selenide

## Abstract

Originating from spin and orbital motion, van der Waals (vdW) ferromagnetism has emerged as a significant platform to experimentally access the fundamental physics of magnetism in reduced dimensions, including quantum computing, sensing, and data storage. However, currently, available vdW ferromagnetic materials can be achieved with mechanical exfoliation and low‐temperature operation, which completely limits the monolithic integration of vdW ferromagnets with other functional materials. Nonetheless, the direct synthesis of room‐temperature vdW ferromagnets has not been achieved commercially, owing to the imprecise control of the layer‐by‐layer growth, high‐temperature synthesis, and low yield. To overcome these limitations, herein, an artificial vdW ferromagnetic platform has been reported, which activates the nano‐crystallization and its corresponding ferromagnetism in bulk VSe_2_ via Ar + H_2_S plasma sulfurization. Sweeping the magnetic field, vdW ferromagnetism has been spatially resolved, which experimentally correlates with magnetization reversal behavior and domain pinning effects. Furthermore, nano‐crystallization of VSe_2_ is clearly validated with transmission electron microscopy, energy‐dispersive X‐ray spectroscopy, X‐ray photoelectron spectroscopy, and selected area diffraction analysis. In conclusion, it is envisioned that the artificial vdW ferromagnetic platform can artificially inject the ferromagnetism in bulk vdW VSe_2_, which has not been possible previously.

## Graphical abstract

Artificial room‐temperature vdW ferromagnetism of bulk VSe_2_ has been presented via nano‐crystallization. By constructing the MFM junction with nano‐crystallized VSe_2_, vdW ferromagnetism has been spatially resolved with a ferromagnetic hysteresis curve. Nano‐crystallization of bulk vdW VSe_2_ is also correlatively observed with cross‐sectional analysis. In conclusion, the artificial room‐temperature vdW ferromagnetic platform can offer an extendable platform for vdW ferromagnetic material, which enables the vdW ferromagnets accessible, engineerable, and 3D integrable into vdW heterostructures for previously unachieved.

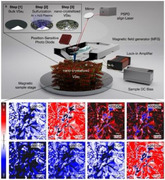

## 1 Introduction

For centuries, the enigmatic properties of lodestones and their magnetic attraction to iron, as well as the remarkable navigational abilities of birds, fish, and insects across vast distances, have captivated human curiosity. Prior to the advent of electromagnetism and quantum mechanics, it was inconceivable that these phenomena might share a common magnetic foundation.^[^ ^1^ ^]^ Magnetism, which is as pervasive as the electron itself, fundamentally originated from the motion and spin of elementary particles. Its applications span living organisms, energy harvesting, data storage, and medical diagnostics. When the microscopic “electron magnets” align spontaneously, magnetic order emerges as a fundamental phase of matter, enabling the development of functional devices such as electric generators, motors, magneto‐resistive memories, and optical isolators. The electron, often regarded as a minute magnet with two opposing poles, generates a magnetic field through its spin and orbital motion. The collective alignment of these microscopic magnets, driven by intrinsic coupling, raises the emergence of ferromagnetism.^[^ ^2^ ^]^ However, the Mermin‐Wagner theorem^[^ ^3^ ^]^ suggested that ferromagnetism cannot be persisted in two‐dimensional (2D) van der Waals (vdW) systems owing to its thermal fluctuations.

Recent breakthroughs in vdW magnetic crystals have demonstrated that magnetic anisotropy can stabilize long‐range magnetic order by creating an excitation gap that counteracts thermal agitation.^[^ ^4^ ^]^ vdW magnetic crystals serve as ideal platforms for exploring magnetism in reduced dimensions,^[^ ^5^ ^]^ offering advantages over traditional magnetic thin films. These materials are largely decoupled from substrates,^[^ ^6^ ^]^ electrically tunable,^[^ ^7^ ^]^ mechanically flexible,^[^ ^8^ ^]^ and amenable to chemical functionalization.^[^ ^9^ ^]^ In early 2017, the first evidence of long‐range magnetic order in pristine vdW crystals was reported in Cr₂Ge₂Te₆ ^[^ ^10^ ^]^ and CrI₃,^[^ ^11^ ^]^ both magnetic insulators with distinct properties. Conversely, vdW Fe₃GeTe₂ ^[^ ^12^ ^]^ was identified as a magnetic conductor, highlighting the diverse applications of itinerant magnets ^[^ ^13^ ^]^ and magnetic insulators.^[^ ^14^ ^]^ Advances in manipulating individual vdW layers have enabled the fabrication of multilayer “designer magnets” ^[^ ^15^ ^]^ with notable outcomes such as giant cross‐layer tunneling magnetoresistance ^[^ ^16^ ^]^ through engineered interlayer magnetic coupling. In heterostructures combining electronic and photonic materials, the integration of distinct physical properties can lead to versatile functionalities, including heterostructure multiferroicity,^[^ ^17^ ^]^ unconventional superconductivity,^[^ ^18^ ^]^ and the quantum anomalous Hall effect.^[^ ^19^ ^]^ Major limitations of vdW ferromagnetic heterostructure correspond to the precise kinetic control of stacking order ^[^ ^20^ ^]^ and large‐scale control over layer thickness ^[^ ^21^ ^]^ and crystallinity.^[^ ^22^ ^]^

To address these challenges, researchers have focused on the extrinsic methods to artificially induce magnetism in non‐ferromagnetic vdW crystals. These approaches include i) defect engineering through vacancies, adatoms, grain boundaries, or edges; ^[^ ^23^ ^]^ ii) substitution of magnetic species; ^[^ ^24^ ^]^ and iii) the magnetic proximity effect,^[^ ^25^ ^]^ where vdW materials are interfaced with magnetic substrates. However, establishing long‐range correlations between extrinsically introduced magnetic moments remains difficult, and substrate‐induced magnetic responses are often limited. Theoretical proposals for inducing ferromagnetism by modifying lattice and band structures have yet to be experimentally realized, arising from the intrinsic vdW lattice structure. In contrast, when ferromagnetism originating from its vdW lattice structure can be experimentally realized, it will drive the versatile advances for vdW ferromagnets ^[^ ^2^ ^]^ and their corresponding applications for electronic,^[^ ^26^ ^]^ spintronics,^[^ ^27^ ^]^ and next‐generation quantum devices.^[^ ^28^ ^]^ These underlying limitations of the conventional vdW ferromagnets motivated us to develop an on‐demand synthesis method of vdW ferromagnet.

Herein, we report an artificial vdW ferromagnetic platform for non‐magnetic vdW crystals, which provides a systematic solution for conventional vdW ferromagnets. To artificially activate the ferromagnetism in bulk vdW VSe_2_, lattice distortion has been conducted with Ar + H_2_S plasma sulfurization, which corresponds to the ion penning effects and ion penetration. The artificial vdW ferroelectricity activates the ferromagnetic manipulation regardless of the number of vdW layers, which comprehensively overcomes the limitations of conventional vdW ferromagnetism. Artificial vdW ferromagnetism was locally observed with Magnetic Force Microscopy (MFM) imaging. The MFM junction was constructed using a Co‐coated magnetic tip, magnetically shielded sample holder, and dual‐permanent‐magnet generator capable of sweeping the in‐plane magnetic field from ‐500 to +500 Oe. The spatial resolution of magnetic domains was achieved by decoupling topography and magnetic force measurements at controlled tip‐sample distances (5 and 25 nm, respectively). ^[^^[^  According to the direction of the magnetic field of the MFM junction, vdW ferromagnetism has been spatially resolved with local ferromagnetic domain mapping of nano‐crystallized VSe_2_, which experimentally correlates with magnetization reversal behavior and domain pinning effects. Furthermore, nano‐crystallization of VSe_2_ was clearly validated with atomic force microscopy (AFM), transmission electron microscopy (TEM), energy‐dispersive X‐ray spectroscopy (EDS), X‐ray photoelectron spectroscopy (XPS), selected area diffraction (SAED) and Raman spectra measurements. In conclusion, we envision that our artificial vdW ferromagnetic platform can artificially manipulate the ferromagnetism in bulk vdW VSe_2_ via nano‐crystallization, which has not been possible previously. Unlike previously reported intrinsic room‐temperature vdW ferromagnets such as Cr_1+x_Te₂,^[^ ^31^ ^]^ Fe₅GeTe₂ ^[^ ^32^ ^]^ and Fe₃GaTe₂,^[^ ^33^ ^]^ which rely on defined magnetic ordering. Nevertheless, critical challenges persist, including intricate magnetic interactions across atomic lattices, scalability limitations in synthetic methodologies, and inherent interfacial inhomogeneity. Our approach enables artificial vdW ferromagnetism via nano‐crystallization in vdW VSe_2_. We envision that this strategy provides the artificial injection of the vdW ferromagnetism, offering the versatile advances in 2D vdW spintronic systems.

## 2 Results and Discussion

### 2.1 Artificial vdW Ferromagnetic Platform for Bulk VSe_2_

**Figure 1:**
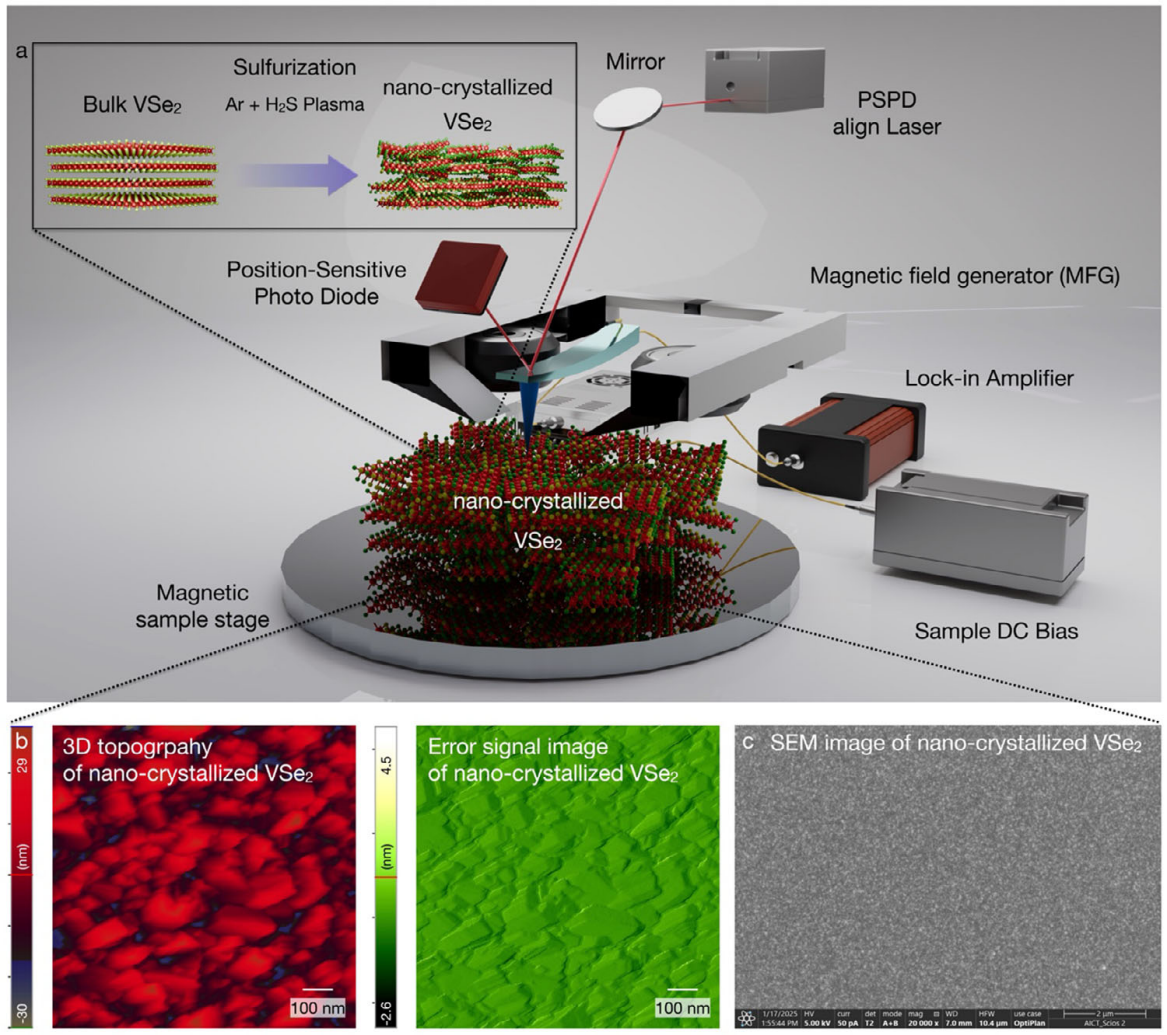
Artificial vdW ferromagnetic platform for bulk VSe_2_. a) Schematic illustration of the artificial vdW ferromagnetic platform, which has been achieved with (i) Ar + H_2_S plasma and (ii) MFM junction. To spatially resolve the ferromagnetism, an MFM junction has been constructed with a magnetic field generator, magnetic sample holder, and Co‐coated magnetic tip, which excludes the generation of the electrical field at the tip‐sample junction. b) AFM image (left side as 3D topography image, right side as error signal image) and c) SEM image of nano‐crystallized VSe_2_.

To artificially induce the ferromagnetism of the bulk vdW 1T‐VSe_2_ (non‐magnetic), single‐step penetrative plasma sulfurization has been utilized to inject the ferromagnetism in bulk vdW VSe_2_ via hydrogen sulfide (H_2_S) + argon (Ar) ion bombardment, which results in the lattice distortion and nano‐crystallization.^[^ ^34^ ^]^ In our previous research, H₂S + Ar ion bombardment generates the nano‐crystallization in van der Waals materials, offering a synthetic platform to isolate the VSe_2_ monolayer for versatile magnetic functionalities. In this study, this approach has been extended to distort the vdW lattice in non‐magnetic bulk VSe₂, enabling the emergence of ferromagnetism through the VSe_2_ monolayer isolation. The monolayer VSe_2_ (1T and 2H phase) is notable for its intrinsic ferromagnetism owing to the strong electron coupling in the 3d^1^ odd‐electronic configuration of V^4+^.^[^ ^30^ ^]^ Astonishingly, the ferromagnetic ordering of monolayer VSe_2_ is robust and persists above room temperature, making monolayer VSe_2_ a significant material for vdW spintronics applications.^[^ ^35^ ^]^ However, the monolayer VSe_2_ is vulnerable to oxidation,^[^ ^21^ ^]^ which vanishes the ferromagnetism of monolayer VSe_2_. Unlike the monolayer VSe_2_, bulk vdW VSe_2_ indicates non‐magnetic properties. Thus, bulk vdW VSe_2_ was intentionally selected for artificial ferromagnetism injection with nano‐crystallization. As shown in **Figure** **1**a, the nano‐crystallized VSe_2_ was fabricated in the following three steps. First, bulk vdW VSe_2_ was mechanically exfoliated and transferred onto a SiO_2_/Si wafer. Second, single‐step penetrative plasma sulfurization (RF power 400 W) was conducted to crystallize the bulk vdW VSe_2_. Third, the MFM junction was constructed for nanoscale observation of the magnetic domain distribution, constructed with a Co‐coated magnetic tip (Figure S1, Supporting Information), magnetic sample holder, and magnetic field generator (Figures S2 and S3, Supporting Information), enabling the spatial observation of ferromagnetic characteristics. Also, nano‐crystallization of VSe_2_ has been observed with AFM and SEM imaging. As shown in Figure 1b, nano‐crystallization of VSe_2_ has been experimentally revealed within the AFM 3D topography image and error signal image. Furthermore, an SEM image of nano‐crystallized VSe_2_ correlatively indicates the nanoscale crystallization. The grain size of nano‐crystallized VSe₂ is distributed as a function of RF plasma power (18.2–45.7 nm).^[^ ^36^ ^]^ MFM domain imaging reveals that the magnetic domain size corresponds with the nanograin size, resulting in grain boundaries operating as pinning sites. For the improvement of large‐scale uniformity, inductively coupled plasma (ICP) systems have been utilized to ensure homogeneous ion bombardment over centimeter‐scale substrates.

### 2.2 Cross‐Sectional Observation of Nano‐Crystallized VSe_2_

**Figure 2:**
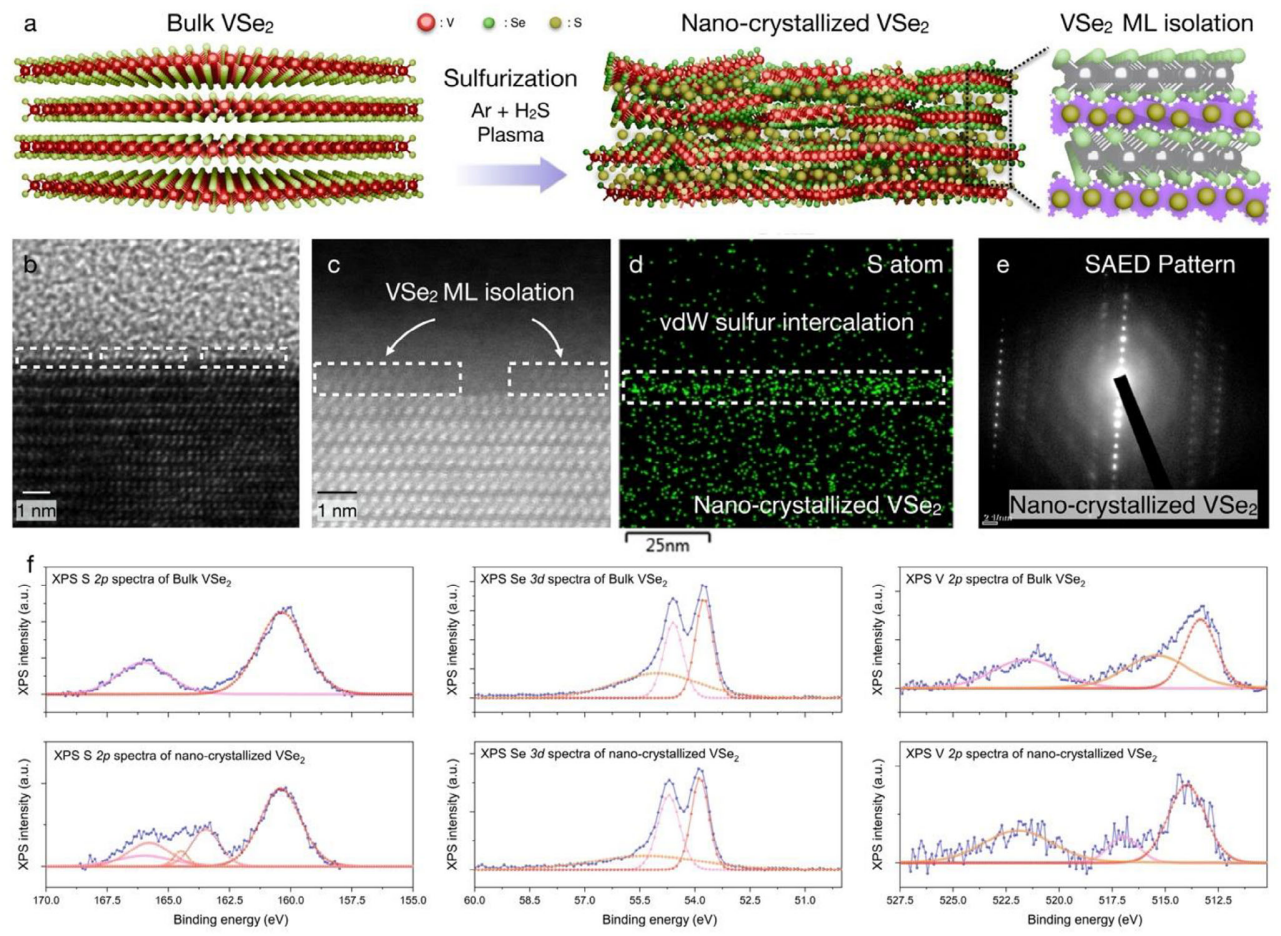
Cross‐sectional observation of nano‐crystallized VSe_2_ via Ar + H_2_S plasma. a) Schematic illustration of nano‐crystallized VSe_2_, generating the VSe_2_ ML isolation. b) Cross‐sectional analysis of nano‐crystallized VSe_2_ with HR‐TEM and STEM image. Cross‐sectional d) EDS mapping and e) SAED patterns of nano‐crystallized VSe_2,_ which is configured with RF plasma 400 W. f) XPS spectra of V *2p*, Se *3d*, S *2p*, exhibiting the sulfurization of bulk vdW VSe_2_ with emergence of V‐S bonding in S *2p* spectra.

Furthermore, cross‐sectional TEM images of nano‐crystallized VSe_2_ were experimentally obtained, exhibiting the VSe_2_ monolayer isolation and its corresponding lattice distortion, as shown in **Figure** **2**a–c. The emergence of room‐temperature ferromagnetism in bulk VSe₂ is attributed to the VSe₂ monolayer isolation via nano‐crystallization. The nano‐crystallization process, driven by local lattice strain during the plasma sulfurization, disrupts interlayer coherence and effectively decouples adjacent VSe₂ layers. This structural decoupling yields electronically quasi‐2D regions where ferromagnetism prevails, thereby facilitating ferromagnetic ordering via Stoner‐type instability. Concurrently, the localized symmetry breaking enhances orbital contributions to magnetism, further enhancing and stabilizing the ferromagnetic behavior. Collectively, these effects enable the realization of robust ferromagnetism at room temperature in an otherwise non‐magnetic bulk VSe₂ system. Cross‐sectional EDS mapping and SAED pattern were correlated to nano‐crystallization. As Bulk vdW VSe_2_ corresponds to Figure S4 (Supporting Information), Figure 2d indicates the nano‐crystallized VSe_2_, comparing the effects of nano‐crystallization. While the EDS mapping indicates the intrinsic distribution of the V atom, Se atom, C atom, and S atom in bulk states, the distribution of the S atom has been dominantly generated at the surface after the nano‐crystallization. As plasma sulfurization is configured with Ar + H_2_S plasma, resulting in the amorphous phase of Bulk vdW VSe_2_ and nano‐crystallization. The SAED pattern of 1T‐ VSe_2_ indicates the periodic pattern, while the nano‐crystallized VSe_2_ lattice (Figure 2e) experimentally clarifies the origin of the observed unidirectional lattice,^[^ ^37^ ^]^ which corresponds to the lattice distortion. XPS measurements were performed to clarify and elucidate variations of chemical bonding within nano‐crystallization, as shown in Figure 2f. As the V *2p* XPS spectra of pristine VSe_2_ have been deconvoluted into the binding energy of 513.3 eV peak, 521.6, and 515.4 eV, each peak has been shifted as 517.0, 514.0, 521.9 eV with nano‐crystallization and its corresponding V‐S bonding formation. Also, S *2p* XPS spectra of pristine VSe_2_ can be separated as 166.0 eV peak and 160.4 eV peak. After crystallization, an additional deconvoluted S *2p* peak has been generated as 164.5, 163.5, and 165.8 eV. Thus, V─S bonding formation and nano‐crystallization can be further clarified with a comparison of S *2p* spectra. Se *3d* peak also exhibits heterogeneity in chemical composition, which can be deconvoluted as 54.6, 53.8, and 55 eV peaks. And Se *3d* peak has been shifted to 54.7, 53.9, and 55.3 eV, which directly corresponds to the Se atom termination with sulfurization. Owing to the ion penning effects, Se atoms were locally terminated by S atoms via plasma sulfurization, which resulted in a substantial increase in the S/Se atomic concentration ratio. The Se‐to‐S substitution ratio was quantified from XPS atomic concentration analysis, yielding sulfur incorporation of ≈11.45 at%, while selenium incorporation exhibits ≈7.30 at% at the nano‐crystallized VSe₂ (400 W). In our sulfurization system, the sufficient generation of H_2_S^+^ for bombardment on VSe_2_ to crystallize the lattice is most significant. The proposed mechanism of H_2_S^+^ generation in this system is the Penning effect by the Ar gas and the direct ionization of H_2_S as follows Equations (1) and (2).^[^ ^38^ ^]^

(1) $$\mathrm{Ar}+e^{-}\to Ar^{+}+2e^{-}$$

(2) $$Ar^{+}+H_{2}S\to \mathrm{Ar}+H_{2}S^{+}$$

Sufficient electrons in the plasma system can directly ionize H_2_S gas to generate H_2_S^+^, as shown in Equation (3). But except for Ar gas, it is difficult to generate H_2_S plasma owing to the low ionization energy characteristics of H_2_S gas.^[^ ^39^ ^]^ To prevent plasma‐induced over‐etching and maintain the integrity of the layered VSe₂ structure, plasma conditions were carefully optimized. The gas mixture was fixed at a 1:1 ratio of Ar and H₂S (50 sccm each), and RF plasma power was set at 400 W. These conditions were found to be optimal for inducing nano‐crystallization without significant damage to the surface or delamination. Preliminary experiments with higher RF power (>500 W) or an Ar‐rich environment resulted in structural degradation and amorphization.

(3) $$e^{-}+H_{2}S^{+}\to 2e^{-}$$

For the precise control of plasma‐based sulfurization, the gas mixture ratio was fixed as Ar and H_2_S as 1:1 (50 sccm injection for each gas). In the bulk plasma, multi‐chain reactions (Equations 1–, 3) lead to an increase in plasma potential, while the substrate remains grounded. This creates a self‐biased system that induces an electric field (sheath region), accelerating positively charged ions (H₂S⁺ and Ar⁺) toward the substrate and resulting in ion bombardment. This simultaneous ion bombardment on the single‐crystal VSe₂ surface induces lattice distortion, breaking the long‐range crystallinity and transforming the surface into a nanoscale, discontinuous crystalline structure. With continued ion exposure, the bombardment penetrates deeper, affecting several vdW layers beneath the surface. Consequently, a few upper layers become nanocrystalline and structurally disordered, while the underlying bulk retains its pristine single‐crystalline structure. This VSe₂ monolayer isolation exhibits emergent ferromagnetic behavior, in contrast to the non‐ferromagnetic nature of pristine single‐crystalline VSe₂. The concurrent Ar⁺ and H₂S⁺ ion bombardment onto single‐crystalline VSe₂ leads to a high density of nanoscale defects, which disrupts the long‐range order and results in nanoscale discontinuous domains of VSe₂. This ion‐induced lattice distortion promotes the formation of nanocrystalline structures (Figure S5, Supporting Information). Similar nano‐crystallization phenomena have been observed in various systems, including wafer‐scale MoS₂–WS₂ vertical heterostructures,^[^ ^40^ ^]^ WS₂–graphene interfaces,^[^ ^6^ ^]^ and other TMDC‐based heterostructures through plasma‐enhanced chemical vapor deposition (PECVD).

### 2.3 Spatially‐Resolved Ferromagnetic Behavior of Nano‐Crystallized VSe_2_

**Figure 3:**
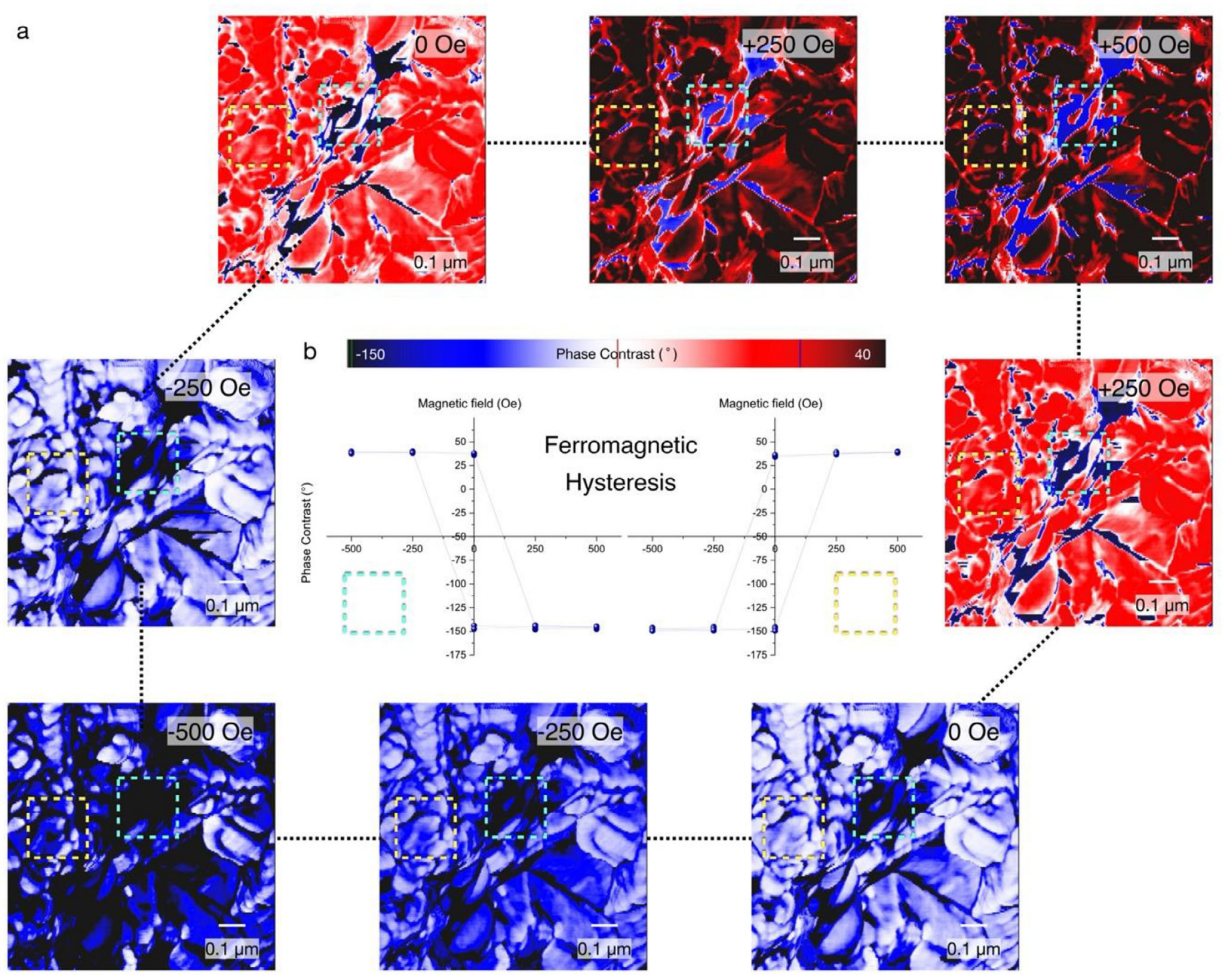
Spatially‐resolved ferromagnetism of nano‐crystallized VSe_2_. Resolving the nanoscale magnetic heterogeneity and ferromagnetic hysteresis behavior via magnetic domain imaging. a) sequential MFM phase images of nano‐crystallized VSe_2_, which has been generated by the magnetic field control. b) Ferromagnetic hysteresis mapping with nanoscale magnetic domain extraction from the selected yellow box (left) and white box (right).

To observe the artificially generated ferromagnetic domain, MFM image has been correlatively conducted with i) magnetic imaging and ii) topography imaging, which allows the observation of long‐range magnetic interactions while minimizing the influence of the topography. While the topography imaging was conducted within a tip‐sample distance of ≈5 nm, which affects the vdW interaction, magnetic force can be clearly obtained with a tip‐sample distance of ≈25 nm. For magnetic field sweep, a magnetic field generator has been attached to control the magnetic field at the MFM junction. A magnetic field generator was constructed with two permanent magnets. With a fixed permanent magnet, the rotation of another permanent magnet locally induces an in‐plane magnetic field at the tip apex between the soft iron localizers. Rotating the permanent magnet results in a controllable magnetic field between the minimum magnetic field (−500 Oe) and the maximum magnetic field (+500 Oe). The magnetic field can be controlled at the tip‐sample junction, the magnetic hysteresis can be spatially resolved, as shown in **Figure** **3**a. As the magnetic field was controlled within 250 Oe duration, the yellow dash box MFM phase images sequentially resolved as ‐147.46° (‐500 Oe), ‐148.21° (‐250 Oe), ‐147.43° (0 Oe), 37.95° (+250 Oe), 39.10° (+500 Oe), 38.96° (+500 Oe), 37.01° (+250 Oe), 34.88° (0 Oe), ‐146.90° (‐250 Oe), ‐149.0° (‐500 Oe). In contrast, the white dashed box in the MFM phase image can be mapped as 38.90° (‐500 Oe), ‐39.28° (‐250 Oe), ‐37.16° (0 Oe), ‐144.63° (+250 Oe), ‐146.72° (+500 Oe), ‐145.18° (+500 Oe), ‐145.94° (+250 Oe), ‐145.16° (0 Oe), 38.55° (‐250 Oe), 39.04° (‐500 Oe). Furthermore, the ferromagnetic hysteresis curve has been mapped from spatially extracted ferromagnetic phase value from each dashed box, as shown in Figure 3b. Owing to the MFM phase mapping, the nanoscale ferromagnetic domain has been experimentally observed with room‐temperature ferromagnetic hysteresis behavior. Regarding the emergence of room‐temperature ferromagnetism in bulk VSe₂ upon Ar + H₂S plasma treatment, the synergistic mechanism has been attributed to i) orbital magnetism and ii) vdW sulfur intercalation, which effectively isolates monolayer VSe₂ regions at the bulk VSe₂ crystal. First, the nano‐crystallization process introduces substantial local strain and atomic disorder into the VSe₂ lattice. These structural perturbations break the spatial inversion symmetry and promote the localization of vanadium 3d orbitals, thereby enhancing orbital magnetic moments. Such orbital contributions are known to play a critical role in transition metal dichalcogenide systems under reduced symmetry, enabling finite magnetization even in the absence of conventional spin ordering. Density functional theory (DFT) calculations on disordered VSe₂ supercells further support this scenario, indicating non‐zero net magnetic moments arising from asymmetric d‐orbital occupancy.^[^ ^41^ ^]^ Second, the incorporation of sulfur atoms during H₂S plasma exposure induces the intercalation of sulfur species into the vdW gaps between VSe₂ layers. This interlayer sulfur intercalation disrupts the electronic coherence along the c‐axis, effectively decoupling adjacent VSe₂ layers and inducing quasi‐monolayer behavior within localized crystalline domains. Previous theoretical and experimental studies have established that monolayer VSe₂ favors itinerant ferromagnetism due to Stoner‐type instability at the Fermi level,^[^ ^42^ ^]^ a behavior suppressed in the bulk form due to strong interlayer hybridization. Our results suggest that the plasma‐induced sulfur intercalation restores the monolayer‐like electronic structure within nano‐crystallized grains, enabling ferromagnetic ordering at room temperature. Thus, the emergence of room‐temperature ferromagnetism in sulfurized VSe₂ arises from a cooperative interplay between orbital magnetism driven by lattice disorder and monolayer isolation effects. This post‐synthetic mechanism represents a novel pathway to modulate the ferromagnetic order in otherwise non‐magnetic vdW materials.

### 2.4 Nano‐Crystallization Effects in the Artificially Generated Ferromagnetic Domain

**Figure 4:**
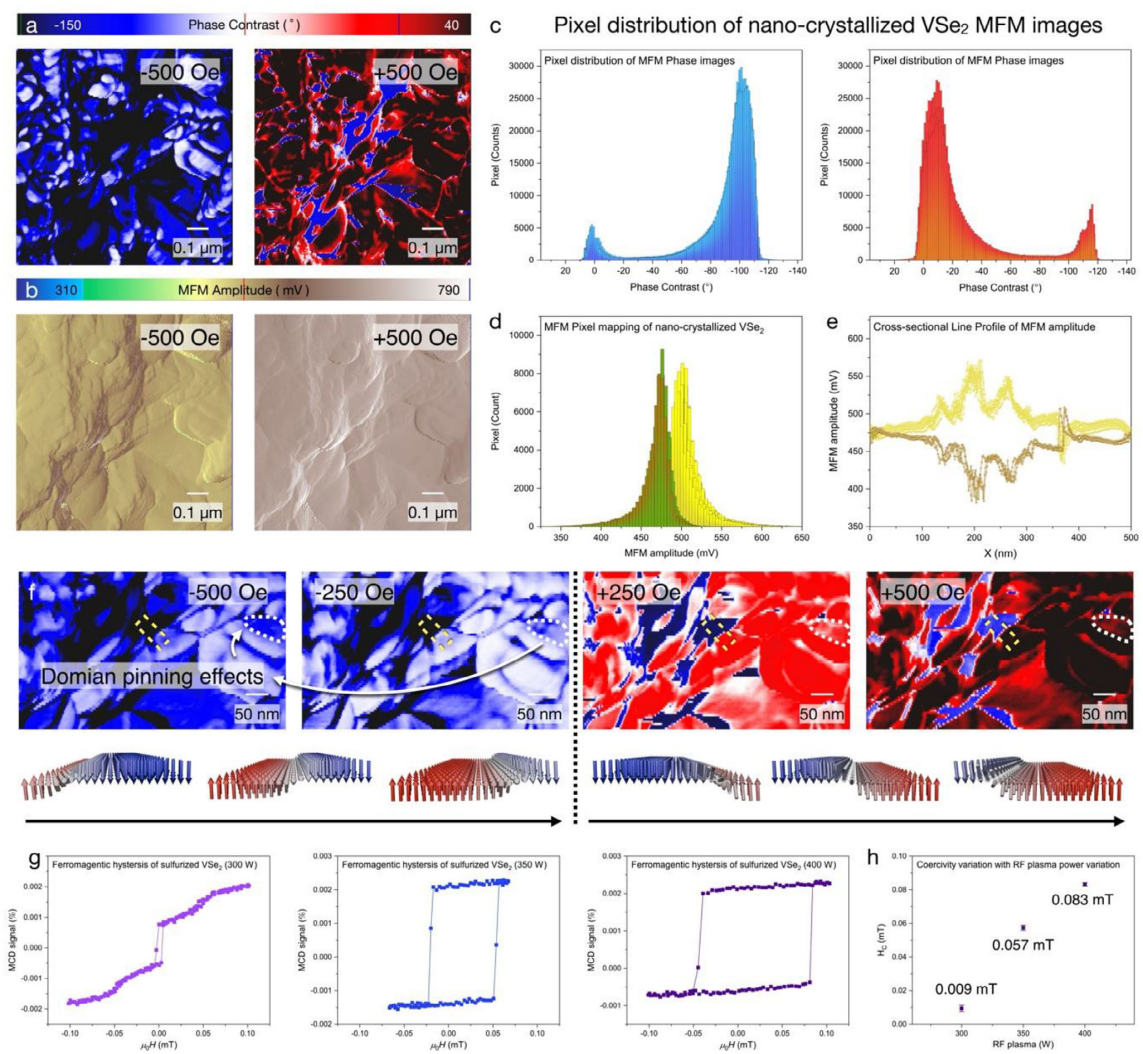
Reversible magnetization of nano‐crystalline ferromagnetic domain. a) MFM phase images (top) and b) MFM amplitude images (bottom) with a heterogeneous magnetic field. Pixel distribution of nano‐crystallized VSe_2_, consisted of c) MFM phase and d) MFM amplitude. e) Cross‐sectional line profiles of MFM amplitude, which indicates magnetization reversal of nano‐crystallized VSe_2_. f) Sequential domain dynamics with magnetic field sweep, resulting in the magnetization reversal of the ferromagnetic domain. g) ferromagnetic hysteresis with RF plasma power variation and its corresponding h) coercivity.

Within the magnetic field weep, MFM phase (**Figure** **4**a) and MFM amplitude (Figure 4b) from opposite magnetic fields (+500 Oe, ‐500 Oe) correspond to the magnetization reversal, which fully operates as a reversible magnet. Furthermore, statistical analysis with nano‐crystallized VSe_2_ MFM pixel distribution has been conducted, as shown in Figure 4c,d.

As shown in Figure 4c, the dominant pixel peak from +500 Oe corresponds to the MFM phase as ‐101.89°, while the dominant pixel peak with −500 Oe has been extracted as 1.36°. Additionally, MFM amplitude pixel mapping exhibits heterogeneous peak distribution of 505.67° and 476.45°, correlating the existence of the heterogeneous magnetic pole (Figure 4d). Such reversible pixel distribution from MFM phase and MFM amplitude images directly supports the magnetization reversal. The cross‐sectional line profile of nano‐crystallized VSe_2_ corresponds to the magnetization reversal within magnetic field control (Figure 4e). To verify that the nano‐crystallization effects for ferromagnetism, magnetization reversal (Figure S6, Supporting Information), and domain pinning effects were also resolved with MFM amplitude images (Figure 4f). Within the maximum magnetic field (+500 Oe, ‐500 Oe), domain distribution of +250 Oe, ‐250 Oe has been expanded owing to the atomic‐scale defects from nano‐crystallization, which induces the domain pinning effects. The emergence of the nanoscale ferromagnetic domain can be explained with nano‐crystallization. When pinning effects are negligible, magnetic domain walls can be spatially shifted and expanded without significant domain wall resistance, leading to high initial magnetization within a minimum external magnetic field. However, the existence of defects enhances the energy barrier of ferromagnetic domain wall movements, which completely blocks the spatial domain wall movements. Regarding the high defect density of nano‐crystallized VSe_2_, the magnetization rises gradually until the external magnetic field is as large as the pinning energy. To further validate the room‐temperature ferromagnetism of nano‐crystallized VSe_2_, bulk VSe_2_ has been investigated with MFM imaging and its corresponding pixel distribution mapping (Figures S7–S10, Supporting Information). As bulk VSe_2_ exhibits non‐magnetic properties, magnetization reversal, ferromagnetic hysteresis, and domain pinning effects were not experimentally observed, unlike nano‐crystallized VSe2. To verify the room‐temperature ferromagnetism observed via MFM, superconducting quantum interference device (SQUID) magnetometry was performed on nano‐crystallized VSe₂. As shown in Figure 4g, the M–H loop at 300 K demonstrates the enlargement of ferromagnetic hysteresis and its corresponding coercivity as ≈0.009 mT (300 W), 0.057 mT (350 W), and 0.083 mT (400 W). Plasma‐dependent coercivity further indicates a nano‐crystallization of bulk VSe2, substantiating the room‐temperature ferromagnetic behavior in our system.

## 3 Conclusion

In conclusion, an artificial room‐temperature vdW ferromagnetism has been achieved in VSe_2_ through nano‐crystallization with plasma sulfurization. Owing to the ion bombardment, the Se atom is randomly terminated to the S atom, resulting in the VSe_2_ monolayer isolation and local lattice distortion. By constructing the MFM junction with nano‐crystallized VSe2, vdW ferromagnetism, and its corresponding ferromagnetic hysteresis curve have been spatially resolved. Nano‐crystallization of bulk vdW VSe_2_ was also correlatively observed with cross‐sectional transmission electron microscopy, energy‐dispersive X‐ray spectroscopy, and selected area diffraction analysis. In conclusion, our artificial room‐temperature vdW ferromagnetic platform can offer an extendable platform for vdW ferromagnetic material, which enables the vdW ferromagnets to be accessible, engineerable, and integrable into emergent heterostructures for previously unachieved.

## 4 Experimental Section

### Nano‐Crystallization

The ICP‐type of plasma‐enhanced chemical vapor deposition (ICP‐PECVD) (AFS‐IC6T, Korea) was used for the crystallization of VSe_2_ to induce its amorphous phase. A high vacuum of ≈5 × 10^−5^ in the PECVD chamber was used to evacuate impurities to achieve clean synthesis without other unexpected reactions before plasma treatment for nano‐crystallization. In this study, only the RF plasma power was fixed as 400 W under constant gas conditions, and the argon and H_2_S flows were maintained at 50 SCCM at a pressure of 25 mTorr at room temperature.

### Mechanical Exfoliation and Transfer of vdW VSe_2_

Before mechanical exfoliation and dry transfer, a polydimethylsiloxane stamp was attached to a glass cover. vdW VSe_2_ was mechanically exfoliated from bulk crystals (HQ Graphene, Netherlands) onto polydimethylsiloxane stamps and then transferred onto the substrate by applying a transfer condition of 70 °C.

### Magnetic Force Microscopy

MFM (NX‐10 AFM, Park Systems, Republic of Korea) was conducted with an MFMR cantilever. The MFMR cantilever was calibrated with a tip radius of 25 nm, a length of 225 µm, a height of 15 µm, a width of 28 µm, and a spring constant of 2.8 N/m, resulting in a resonance frequency of 75 kHz. A magnetic sample holder was loaded to block the electrical field generation at the MFM junction. Additionally, a magnetic field generator was further attached to artificially induce the magnetic field and observe the magnetic hysteresis, which was spatially resolved at the MFM junction. Before the MFM measurements, the MFM tip was magnetized as an “N” pole for 15 min.

### Material Characterization

XPS measurements (XIS Supra+, Kratos, United Kingdom) were used to characterize VSe_2_, with an X‐ray spot size of 400 µm. Peak deconvolution was performed on the V 2*p*, Se 2*p*, and S 2*p* signals, with the profiles aligned using the C 1s peak at 285 eV. The XPS data were calibrated using the CASAXPS software (version 8.1). Optical microscopy (U‐MSSP4, Olympus, Japan) and FE‐SEM (S‐4800, Hitachi, Japan) were used to examine the transferred flakes. For cross‐sectional TEM specimen preparation, a focused ion beam instrument (NX2000, Hitachi Ltd., Japan) was used, employing a Ga^+^ ion beam (30–5 keV) and a lift‐off process to etch the specimens. TEM (JEM‐2100F, JEOL, Japan) and XRD (Empyrean, Malvern PANalytical, United Kingdom) were used to observe the lattice structure, EDS, and SAED patterns of the layered VSe_2_ structures at the atomic scale.

## Conflict of Interest

The authors declare no conflict of interest.

## Author Contributions

J.L., G.K., H.S., H.C. contributed equally to this work. J.L., G.K., H.S., and H.C. prepared samples and performed experiments. H.L., S.L., G.K., H.K., S.S., S.S., and D.L. performed the technical discussions on plasma sulfurization. H.S., S.H., G.B., H.H. conducted the analytical experiments, including TEM, EDS, and XPS measurements. G.H., G.J., and Y.L. provided technical advice on the MFM system and magnetic field generator. T.T. and K.W. provide the bulk hexagonal boron nitride samples. A.O., Y.K., and L.F. A.H., W.J., W. S., S.C., H.K., J.L., G.K., H.S., H.C. and T.K. wrote the manuscript with contributions from all the authors. T.K. designed and supervised the study. All the authors have read and approved the final version of this manuscript.

## Supporting information



Supporting Information

## Data Availability

The data that support the findings of this study are available from the corresponding author upon reasonable request.
