# Supplementary material for: Artificial Room‐Temperature Ferromagnetism of Bulk van der Waals VSe2
Source: Adv Sci (Weinh). 2025 May 30;12(34):e04746. doi: 10.1002/advs.202504746 (PMC12442629; doi:10.1002/advs.202504746)
Supplement: Supplementary file 1 — Supporting Information [file ADVS-12-e04746-s001.docx]

Supporting Information

Artificial Room-temperature Ferromagnetism of Bulk van der Waals VSe_2_

*Jinhyoung Lee^†^, Gunhyoung Kim^†^, Hyunho Seok^†^, Hyunbin Choi^†^, Hyeonjeong Lee, Seokchan Lee, Geonwook Kim, Hyunho Kim, Seowoo Son, Sihoon Son, Dongho Lee, Hosin Hwang, Hyelim Shin, Sujeong Han, Geumji Back, Alexina Ollier, Yeon-Ji Kim, Lei Fang, Gyuho Han, Goo-Eun Jung, Youngi Lee, Hyeong-U Kim, Kenji Watanabe, Takashi Taniguchi, Wonjun Shin, Suraj Cheema, Andreas Heinrich, Won-Jun Jang and Taesung Kim**

**
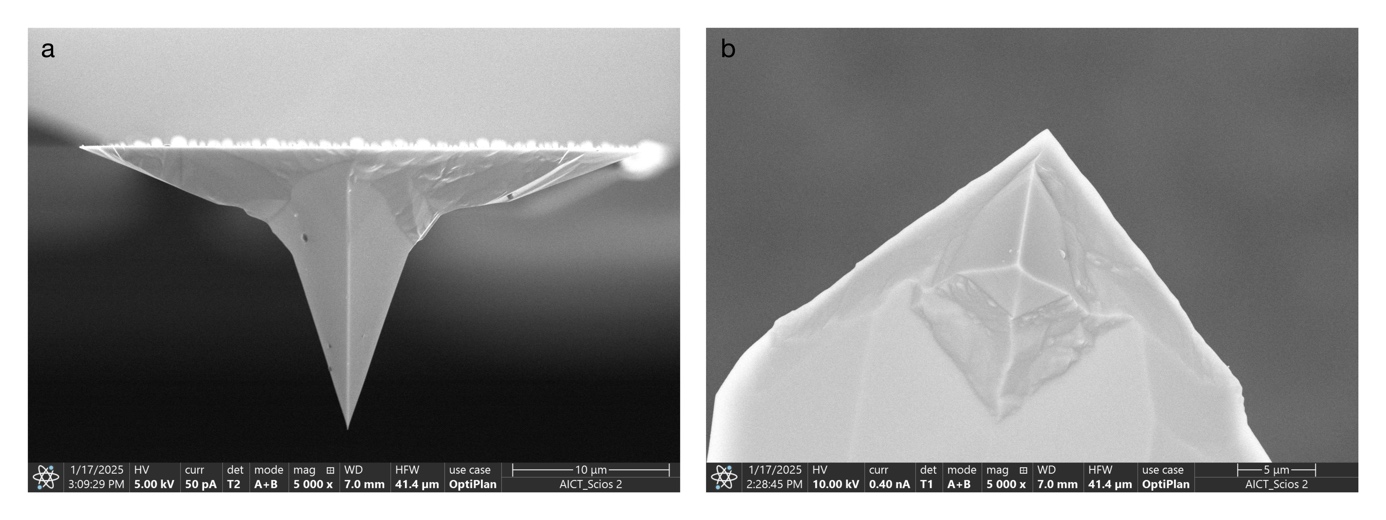
**

**Figure S1. SEM image of the magnetic tip.** a) Front-view SEM image and b) top-view of the Co-coated magnetic tip, which is constructed with a 25 nm tip apex, force constant 2.8 N/m, and coercivity as 300 Oe.

*
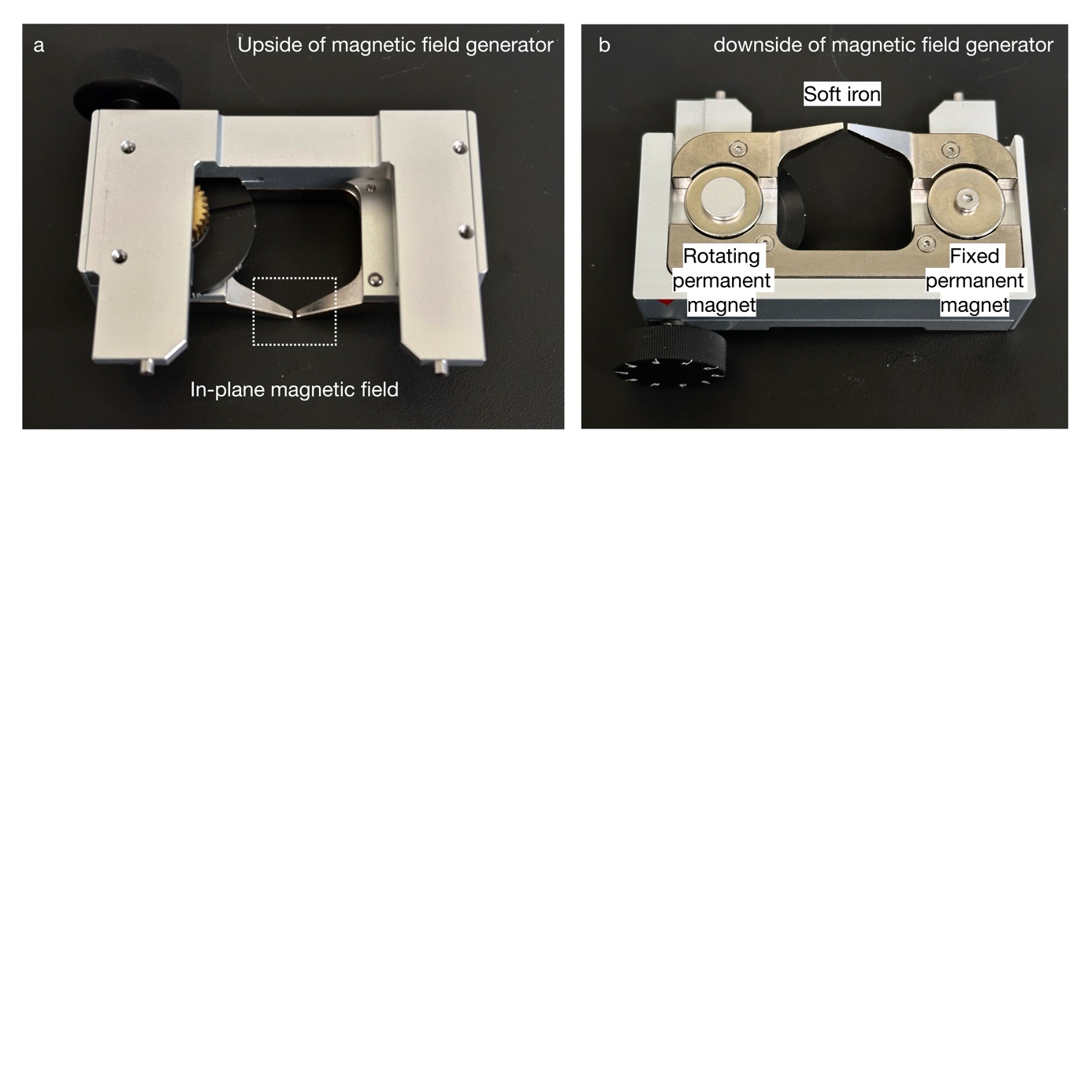
*

**Figure S2. Experimental construction for magnetic field generator.** Photography of a) upside and b) downside of magnetic field generator, which is constructed with two permanent magnets. Rotation of the permanent magnet induces the in-plane magnetic field at the tip-sample junction.

*
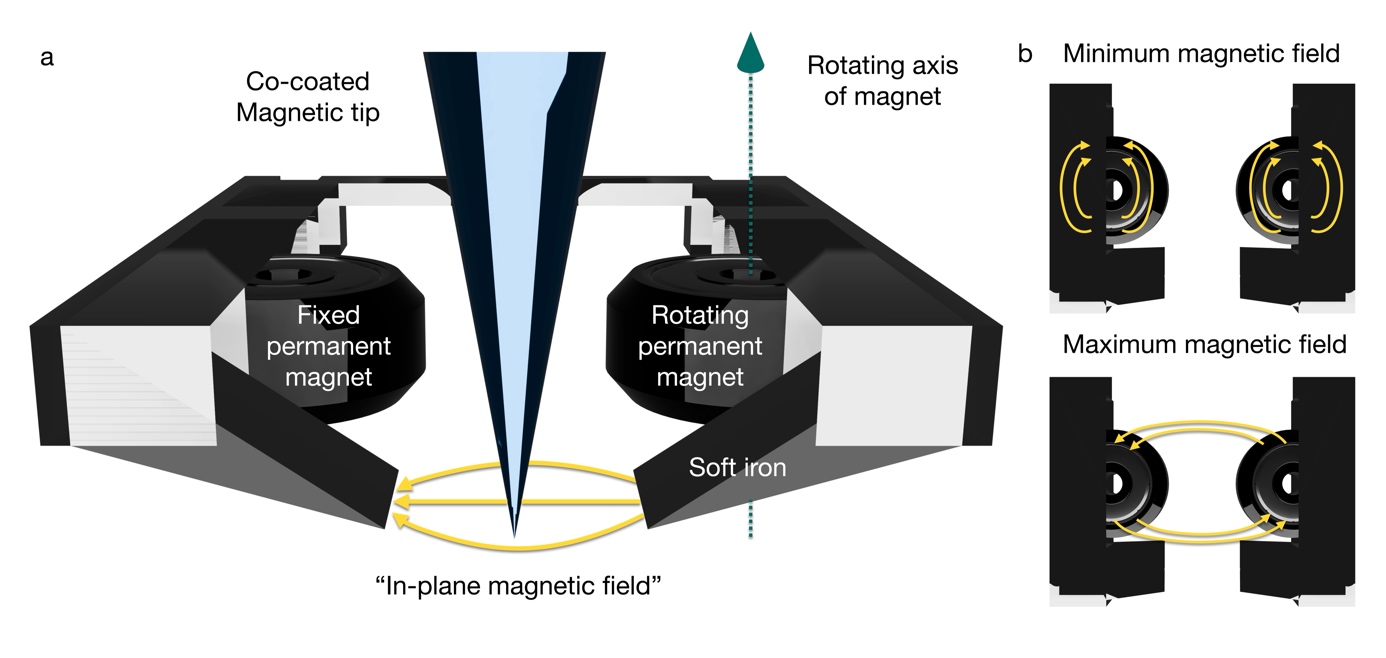
*

**Figure S3. Schematic illustration of magnetic field generator.** a) Schematic illustration of magnetic field generator. Within a fixed permanent magnet, the rotation of another permanent magnet induces the in-plane magnetic field. b) Comparison of minimum magnetic field and maximum magnetic field within two permanent magnet systems.


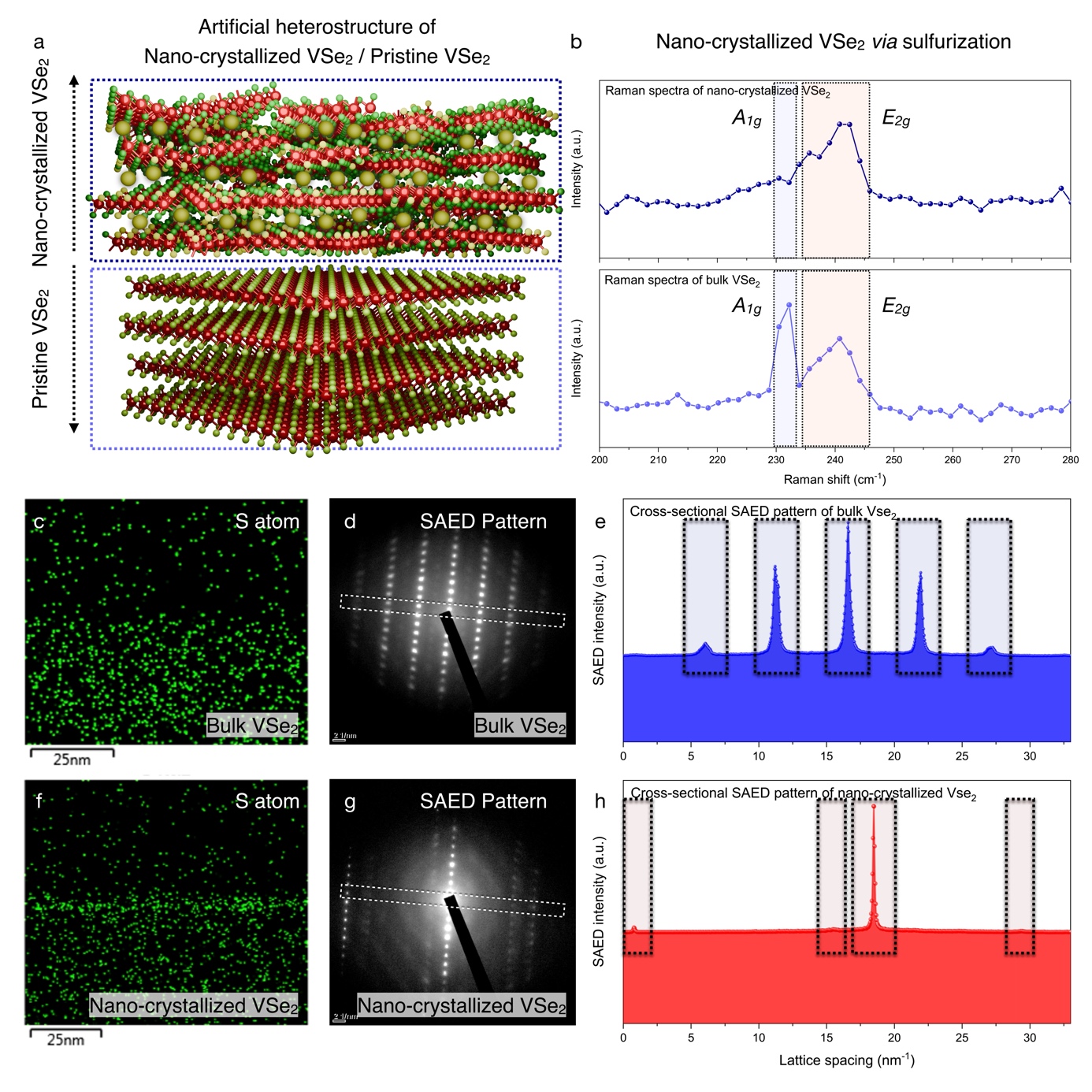


**Figure S4. Nano-crystallization of vdW VSe_2_.** a) Schematic illustration of nano-crystalline formation of bulk VSe₂ flake, isolating the VSe_2_ monolayer. b) Raman spectra of pristine VSe_2_ and nano-crystallized VSe_2_, exhibiting the absence of A*_1g_* peak. c) Cross-sectional EDS mapping, d) SAED pattern, and e) SAED line profile of nano-crystallized VSe_2_. f) Cross-sectional EDS mapping, g) SAED pattern, and h) SAED line profile of bulk VSe_2_.


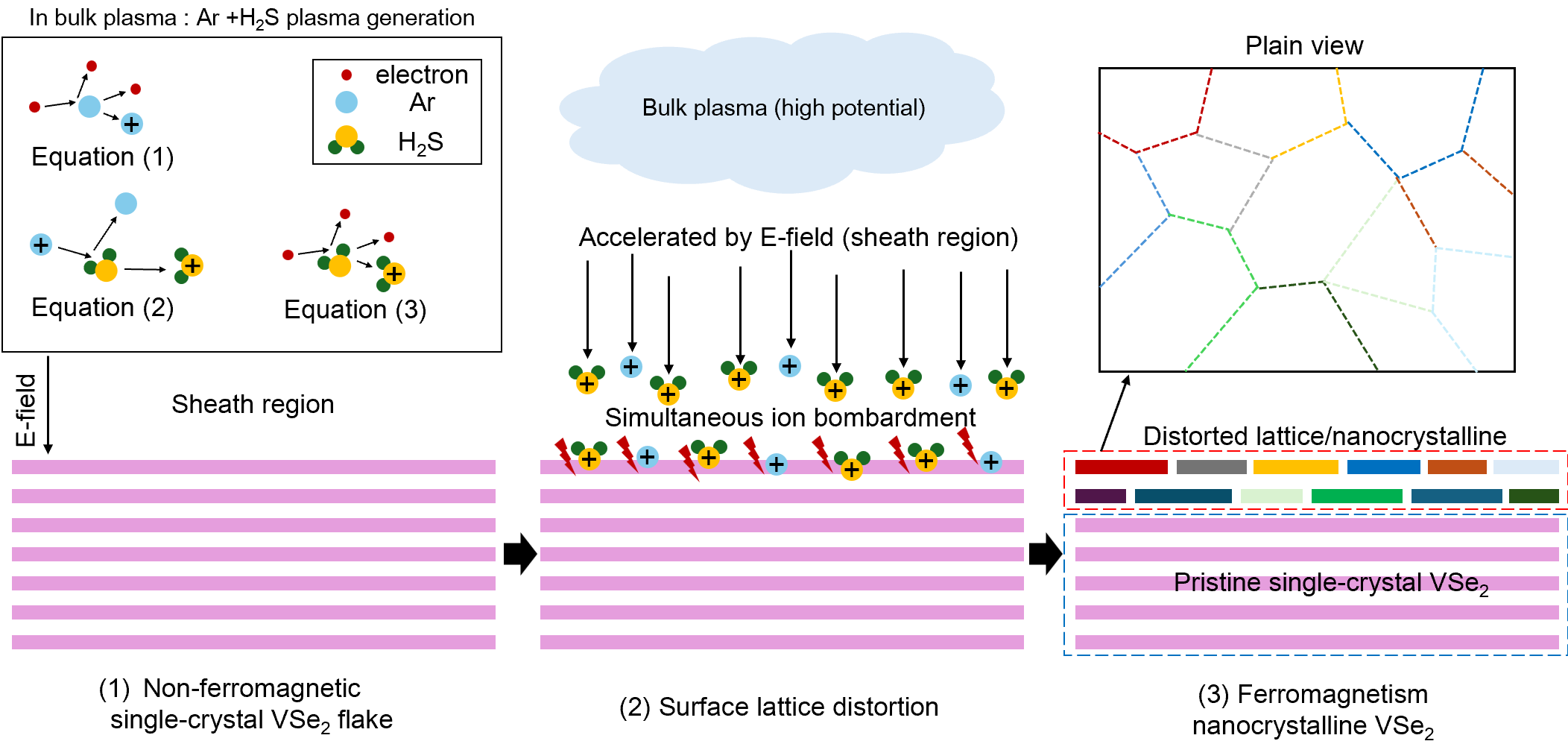


**Figure S5. Mechanism of nano-crystallization of VSe_2_.** Schematic illustration of the lattice distortion and nano-crystalline formation mechanism induced by Ar + H₂S ion bombardment on a bulk VSe₂ flake. The process involves ionized species (H₂S⁺ and Ar⁺) accelerated toward the substrate under the self-biased electric field generated in the plasma sheath region. The resulting ion bombardment disrupts the surface single-crystal lattice, leading to the VSe_2_ monolayer isolation in the upper vdW layers, while the underlying bulk retains its pristine single-crystalline form.


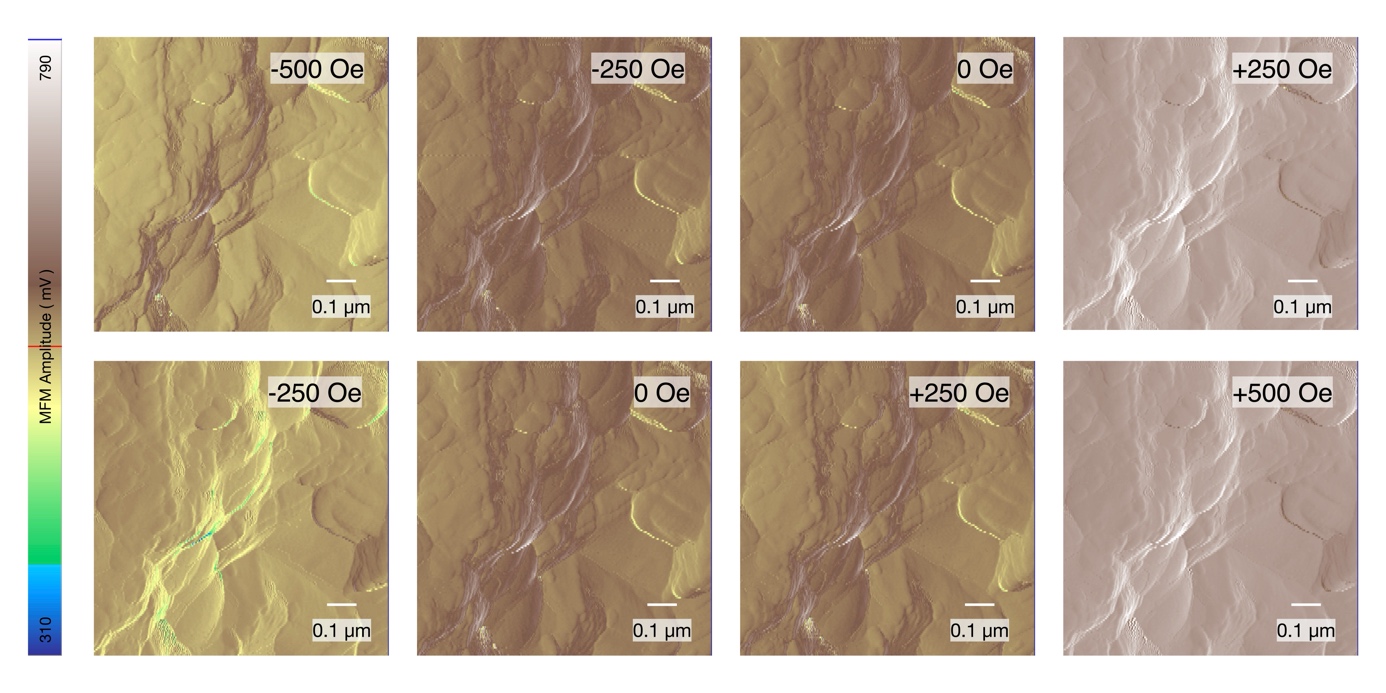


**Figure S6. Observation of magnetization reversal.** Sequential magnetic field sweep for magnetization reversal, which exhibits opposite magnetism distribution at +500 Oe and -500 Oe.

**
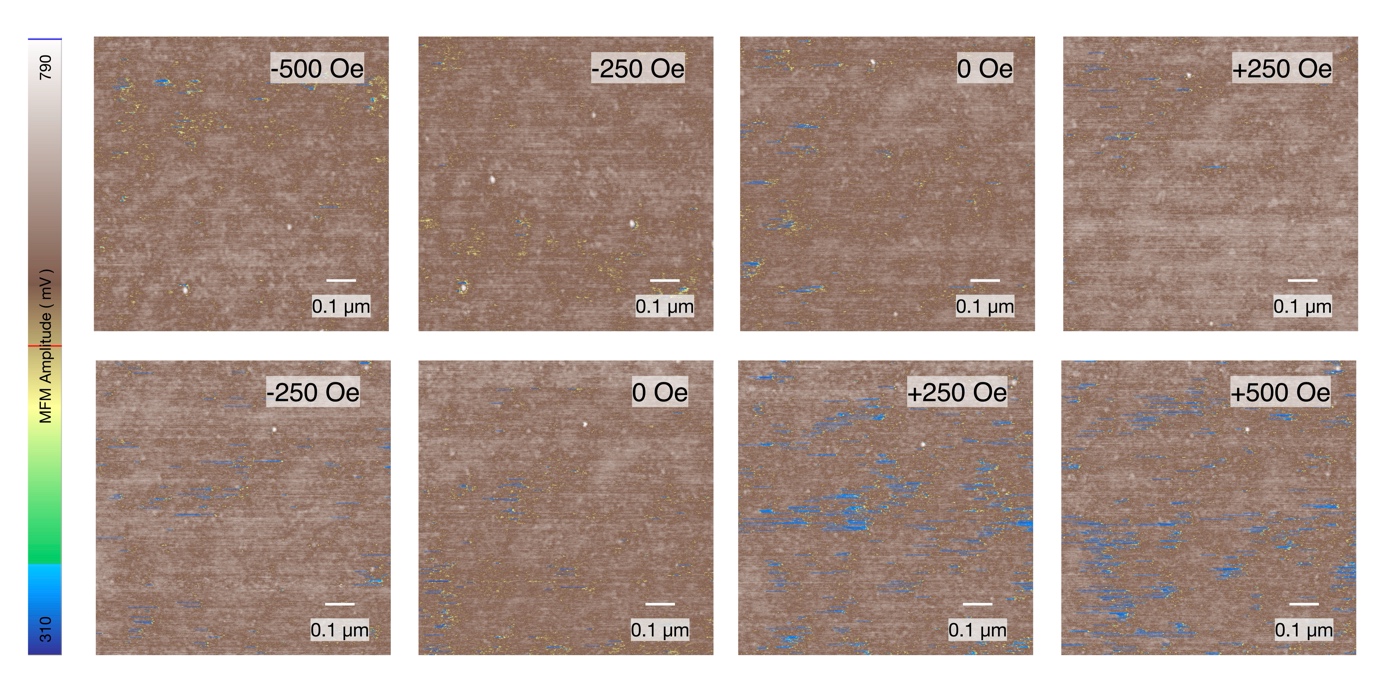
**

**Figure S7. Observation of homogeneous MFM amplitude images.** Sequential magnetic field sweep for non-magnetic VSe_2_, which exhibits homogeneous MFM amplitude mapping at +500 Oe ~ -500 Oe.


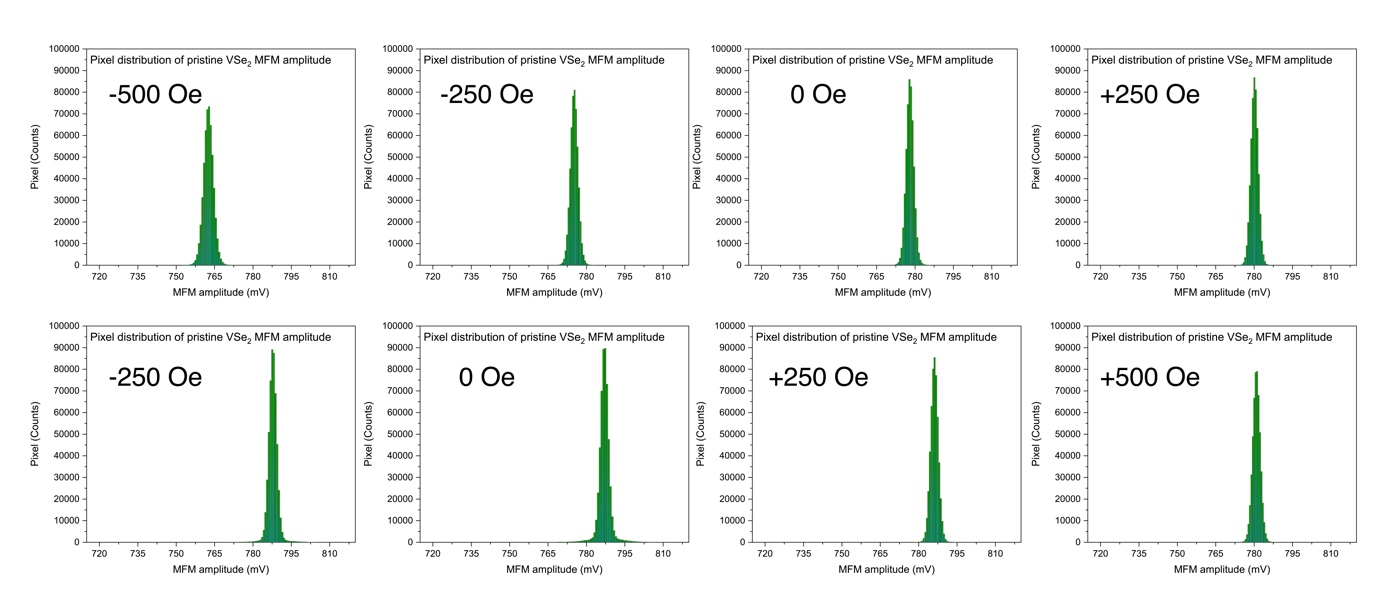


**Figure S8. Pixel distribution of homogeneous MFM amplitude images.** Sequential magnetic field sweep for non-magnetic VSe_2_, which exhibits homogeneous MFM amplitude mapping at +500 Oe ~ -500 Oe.


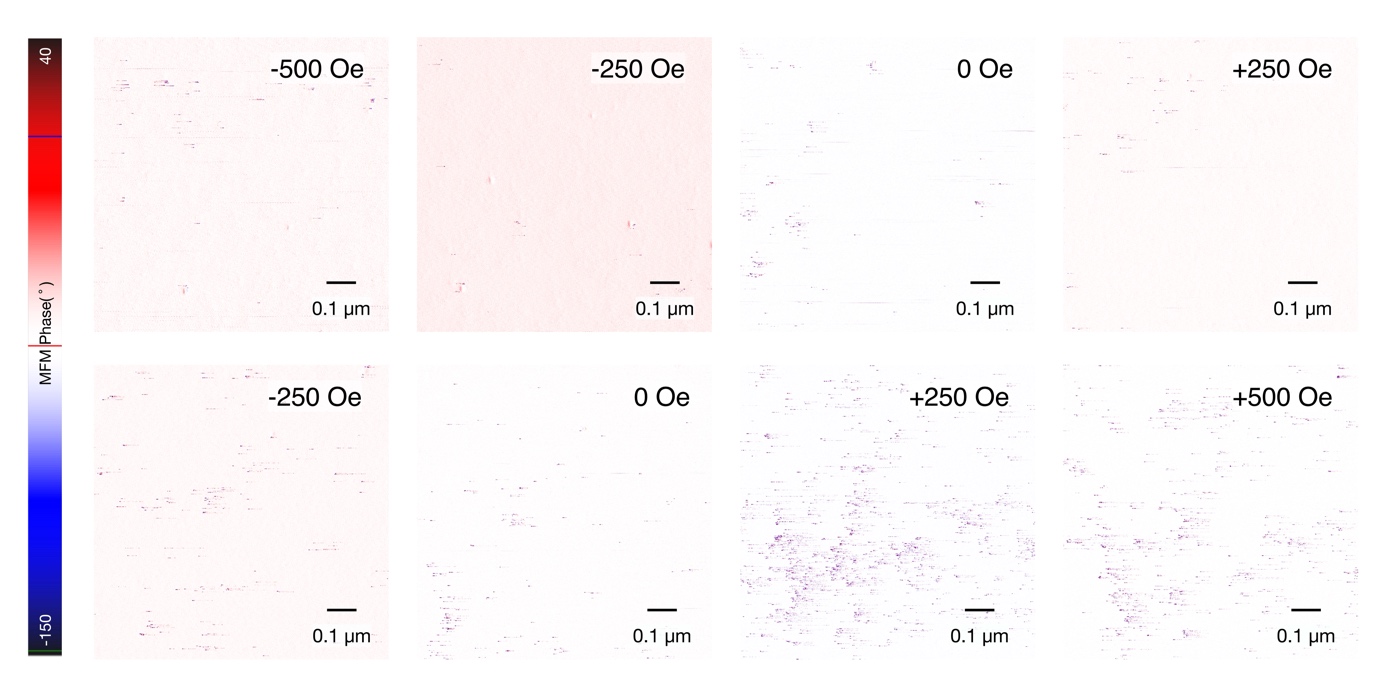


**Figure S9. Observation of homogeneous MFM phase images.** Sequential magnetic field sweep for non-magnetic VSe_2_, which exhibits homogeneous MFM phase mapping at +500 Oe ~ -500 Oe.

**
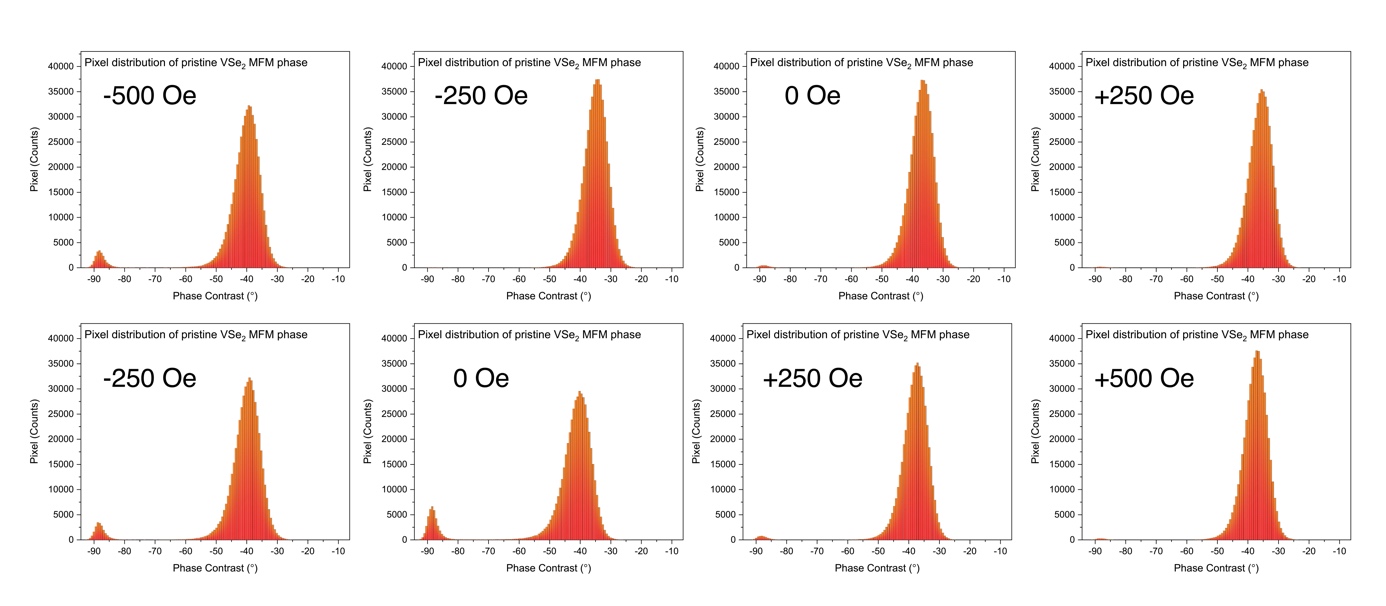
**

**Figure S10. Pixel distribution of homogeneous MFM phase images.** Sequential magnetic field sweep for non-magnetic VSe_2_, which exhibits homogeneous MFM phase mapping at +500 Oe ~ -500 Oe.
